# Experimental Limits of Ghost Diffraction: Popper’s Thought Experiment

**DOI:** 10.1038/s41598-018-31429-y

**Published:** 2018-09-04

**Authors:** Paul-Antoine Moreau, Peter A. Morris, Ermes Toninelli, Thomas Gregory, Reuben S. Aspden, Gabriel Spalding, Robert W. Boyd, Miles J. Padgett

**Affiliations:** 1 School of Physics and Astronomy, University of Glasgow, Glasgow, G12 8QQ UK; 2 Department of Physics, Illinois Wesleyan University, Bloomington, Illinois 61701 USA; 3 Department of Physics, University of Ottawa, Ottawa, Ontario Canada; 4 The Institute of Optics and Department of Physics and Astronomy, University of Rochester, Rochester, NY 14627 USA

## Abstract

Quantum ghost diffraction harnesses quantum correlations to record diffraction or interference features using photons that have never interacted with the diffractive element. By designing an optical system in which the diffraction pattern can be produced by double slits of variable width either through a conventional diffraction scheme or a ghost diffraction scheme, we can explore the transition between the case where ghost diffraction behaves as conventional diffraction and the case where it does not. For conventional diffraction the angular extent increases as the scale of the diffracting object is reduced. By contrast, we show that no matter how small the scale of the diffracting object, the angular extent of the ghost diffraction is limited (by the transverse extent of the spatial correlations between beams). Our study is an experimental realisation of Popper’s thought experiment on the validity of the Copenhagen interpretation of quantum mechanics. We discuss the implication of our results in this context and explain that it is compatible with, but not proof of, the Copenhagen interpretation.

## Introduction

**Figure 1:**
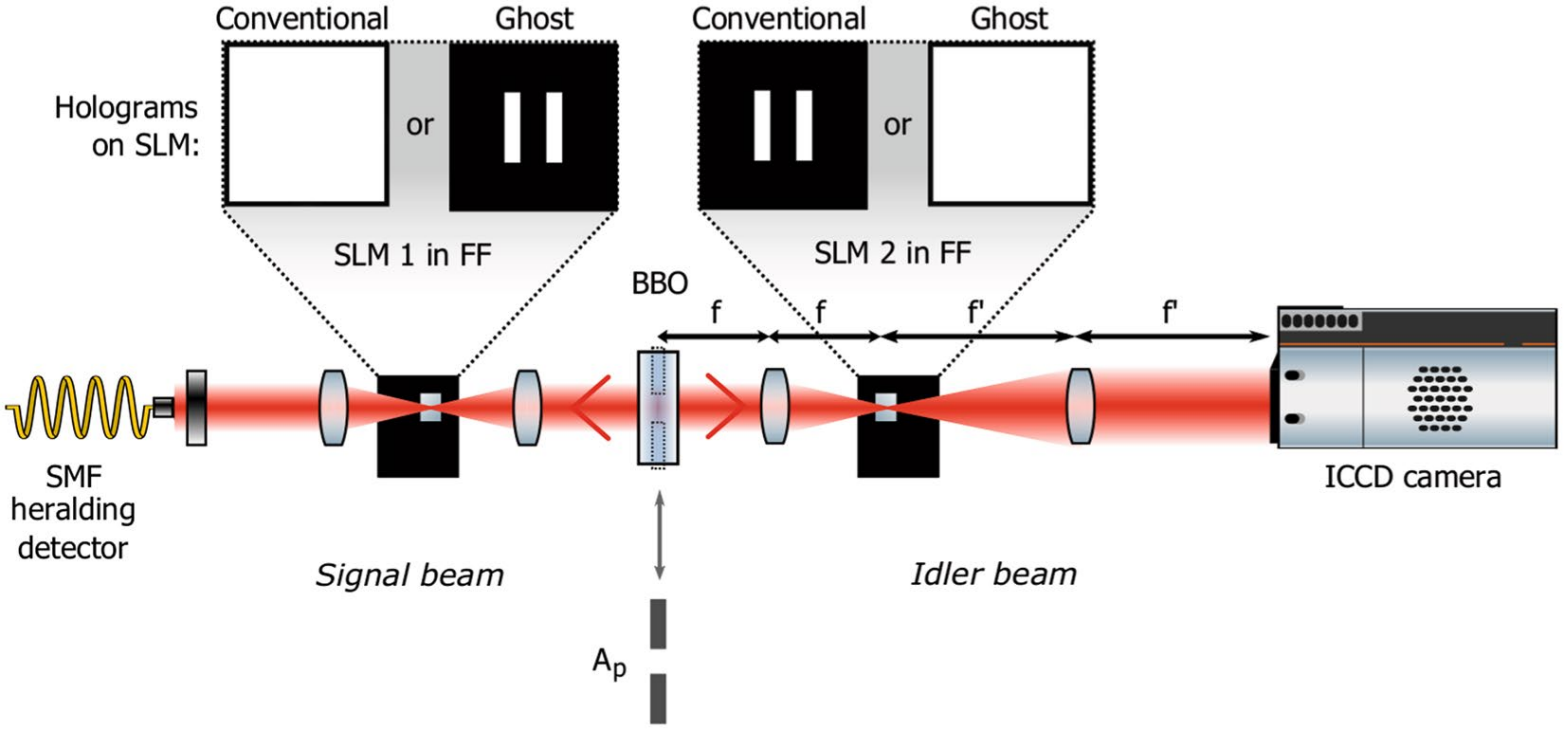
Experimental setup for quantum ghost diffraction and conventional diffraction. A non-linear BBO crystal pumped with a laser at 355 nm generates spontaneous parametric down-converted photon pairs. The two photons are separated and travel in 2 different arms. One of them, the herald, is first collected into a single-mode fibre and is detected by an avalanche photodiode (APD) and the second one is detected by an ICCD camera triggered by the APD. The two photons are reflected from two independent SLMs before being detected. As shown in the figure, the pattern used on each SLM determines if the system is used in conventional or ghost diffraction configuration. Importantly the pump beam size at the crystal can be controlled by introducing an aperture *A*_*p*_ before the crystal, thereby controlling the transverse extent of the spatial correlations between the two generated photons in the far-field of the crystal plane (FF).

Quantum ghost diffraction was introduced together with ghost imaging by Shih and co-workers in 1995^1,2^. These “ghost” experiments refer to configurations where the image or diffraction pattern is obtained from a spatial distribution of photons that have not themselves interacted with the object. Similar to ghost imaging^3^, ghost diffraction and interference were first demonstrated using quantum correlated light and there was initially uncertainty as to whether ghost diffraction was inherently a quantum phenomenon. It was, however, later demonstrated that ghost diffraction can be obtained through the use of classical correlations^4–6^ such as the correlations between two copies of a quasi-thermal light field. In quantum ghost diffraction, correlated photons are generated, separated into two optical arms and then detected by two spatially separated detectors (see Fig. 1). The first of these detectors is an imaging detector that detects the position of the one of the photons that has propagated freely from the source. The second detector, in the other arm, is a single-element, non-spatially resolving detector which detects the photons that are diffracted by the object. The diffraction pattern generated by interaction with the object cannot be measured by either of the detectors alone but rather requires the measurement of the correlation between the two detectors. Typically, in quantum ghost diffraction a spontaneous down-conversion source is used to generate correlated photon pairs, over a wide range of spatial modes. After diffraction of one of the photons by the object, in order to retrieve any diffraction information with maximum contrast, the single-element detector must select only one of the down-converted spatial modes^7^. In the other arm, the other detector spatially resolves the position of the correlated photon (that has not interacted with the object). The correlation between these two detector signals reveals the diffraction pattern of the object.

The importance of ghost diffraction was first pointed out by Shih *et al*. when they established a link between quantum ghost diffraction and Popper’s thought experiment^8^ which Popper thought would challenge the Copenhagen interpretation of quantum mechanics^9,10^. Popper described what we would now refer to as a ghost diffraction experiment, with two EPR correlated particles propagating in two different arms. He argued that a slit placed in one arm should, if the Copenhagen interpretation of quantum mechanics is correct, also cause the photon in the other arm to diffract. Indeed due to position correlations, one can infer the position of the second photon as a consequence of the presence of the slit in the first photon arm. An increase in the knowledge of the photon position should according to Popper’s understanding of the Copenhagen interpretation lead to a spread in its momentum distribution. According to Popper, this remote action would seem either to enable faster-than-light communication or, if the second particle does not actually diffract, challenge the Copenhagen interpretation. Sudbery objected to Popper’s arguments in that the actual transverse extension momentum of an EPR state was infinite and that consequently no further spread in the photon momentum could be measured^11^. Subsequently, Collett and Loudon^12^ showed theoretically that Popper’s arguments actually fail if we consider a realistic source thereby presenting an uncertainty in the position where the two particles are produced: in this case the measured spread in momentum actually never gets bigger than the original spread due to this uncertainty.

Even though previous experimental studies have tried to examine Popper’s objections to the ‘orthodox’ interpretations of quantum mechanics^8,13–15^, the problem remains a topic of open discussion^16^.

Recently we have performed an experimental comparison of the resolution of conventional imaging and ghost imaging^17^. In that work we showed that whilst in the case of conventional imaging the resolution is largely independent of the source characteristics, in the ghost-imaging configuration the resolution of the resulting image is strongly dependent on the transverse extent of the correlations produced by the down-conversion source. In this present work we turn from ghost imaging to ghost diffraction to complete the previously reported experimental implementations of Popper’s ‘Gedankenexperiment’ by a quantitative comparison of conventional and ghost diffraction. We show two asymptotic trends in the observed ghost diffraction. The first trend corresponds to the spread in transverse momentum of the coincidently detected photons within the ghost diffraction while the second trend corresponds to Collett and Loudon theoretical predictions which place an upper limit of the transverse extent of this ghost diffraction pattern. Our demonstration is executed using a systematic comparison between ghost diffraction (when the diffracting object and spatially resolving detector are in different arms) and the conventional diffraction (when the diffracting object and spatially resolving detector are in the same arm) obtained under exactly the same experimental parameters (see Fig. 1). By distinguishing the two trends we unify two visions of the same experiment and show that both the Copenhagen interpretation and causality are in fact perfectly compatible with experimental observations.

For the purpose of the experiment we use double slits of variable slit width to produce the diffraction pattern. Using a double slit rather than a single slit has the advantage of generating a self scaled demonstration. An interference-diffraction pattern of a double-slit is expected to exhibit both a sinusoidal beating due to the interference between the two slits and a sinc envelope due to the diffraction of the single slits. To test Popper’s ideas we have to study the extent of the sinc envelope. According to Popper’s predictions about the experiment^18^ when a slit is placed in one arm, the other particle should not be affected and no diffractive spreading should be recorded for the particle in the other arm. Thus if Popper is correct we would expect the recorded interference patterns to exhibit the same sinc envelope whatever the size of the ghost slits. On the other hand Popper thought that the Copenhagen interpretation would predict that introducing the slit in the first arm should make this second particle diffract more, as long as the presence of the slit makes it possible to infer a more precise position for the particle in the second arm. Under such conditions the prediction is therefore that the recorded diffraction patterns should exhibit a larger sinc envelope as the slit becomes narrower. Collett and Loudon have however emphasised that such a spread would be ultimately limited by the finite size extent of the EPR-like correlations for a realistic implementation and in accordance with both the standard quantum mechanics and the Copenhagen interpretation.

With regards to the interference sine beating expected with a double slit, one expects to obtain similar sinusoidal period for all the recorded interference patterns with no influence from the various experimental condition (slits’ width, either ghost diffraction or conventional diffraction configuration, illumination conditions) as the distance between the two slits is not changed. Thus by using a double slit rather that a single slit one can count the number of fringes appearing in the diffraction pattern to assess the extent of the spread due to the diffraction, thereby generating a self scaled demonstration.

## Results

Within our experiment, the source is a spontaneous parametric down-conversion (SPDC) non-linear crystal producing two correlated light beams, called signal and idler. The essence of the Collett and Loudon argument applied to our situation is then the following. In any down-conversion experiment one sees a spatial distribution of both the signal and idler beams, which in the image plane of the crystal simply corresponds to the width of the pump beam. Placing an aperture in one of the beams (signal) will introduce a diffraction of the beam which may extend the spatial distribution of that beam, but will leave the other beam unaffected. Any pattern obtained from the coincidence detection, or post selection, from this idler beam cannot exhibit a spatial spread exceeding that of the original distribution. For the increase in divergence in the idler beam to be noticeable the diffractive-spreading, *θ* = *λ*/*d*, due to the slit of width, *d*, needs to be greater than the natural divergence of the beam, *α*, arising from the SPDC process for a crystal of length L, i.e.,

1 $$\theta  > \alpha $$

where the phase matching imposes,

2 $$\alpha =\sqrt{\lambda /L}$$

Although the slit can be arbitrarily narrow causing significant diffraction of the signal beam the effective width (i.e. ghost image) of the slit in the idler beam is limited by the finite resolution of the ghost imaging effect. The resolution of any ghost imaging system cannot exceed the transverse correlation length of the spatial correlation between the signal and idler photons^19^, given approximately by

3 $${\sigma }_{c}\gtrsim \frac{1}{2}\sqrt{\lambda L}$$

This resolution limit sets a lower effective size of the ghost image of the slit to

4 $${d}_{{\rm{eff}}}\gtrsim \sqrt{\lambda L}$$

The ghost diffraction is therefore limited i.e.,

5 $$\theta \lesssim \sqrt{\lambda /L}$$

consequently,

6 $$\theta \lesssim \alpha $$

Hence we see that the diffractive effect on the idler of the slit placed in the signal beam does not exceed the idler’s intrinsic divergence. In other words and in the language of the Copenhagen interpretation of quantum mechanics: as long as narrowing the slit placed in the signal beam actually gives an increased knowledge about the position of the correlated idler photons, then these idler photons experience an increase of the spread of their momentum. However, as soon as the non-ideality of the EPR source becomes apparent in that the slit becomes so small that further narrowing it does not yield an increased knowledge of the idler position then no further increase in the spread of their momentum is observed.

A simplified version of the experimental setup is shown in Fig. 1. A non-linear BBO crystal pumped with an UV laser at 355 nm generates spontaneous down-converted (SPDC) photon pairs. The two photons are separated using a pellicle beam splitter into 2 different optical arms. After reflection from an SLM (spatial light modulator), the first photon is collected by a single-mode fibre and detected by a single photon avalanche photodiode (SPAD). Importantly, the spatial selectivity of the single-mode fibre ensures the spatial coherence of the recorded diffraction. After reflection from a second SLM, the second photon travels through an image-preserving delay line (not shown in Fig. 1) before being detected by an ICCD camera triggered by the SPAD upon detection of the first photon. The delay line allows compensation of the camera latency to ensure that the camera is triggered at exactly the right time to capture the photon as it strikes the sensor. As shown in Fig. 1, when the double slits are displayed in the arm containing the single-element detector, the configuration corresponds to a ghost diffraction experiment. Conversely when the double slits are displayed in the camera arm, one can detect conventional, albeit single-photon, diffraction. Moreover, we can optionally restrict the diameter of the pump beam by introducing an aperture *A*_*p*_. Note that, in our experimental setup, we have placed the double slits in the far-field of the down-conversion crystal and that since we observe the diffraction in the far-field of double slits this diffraction plane corresponds to the image plane of the crystal.

**Figure 2:**
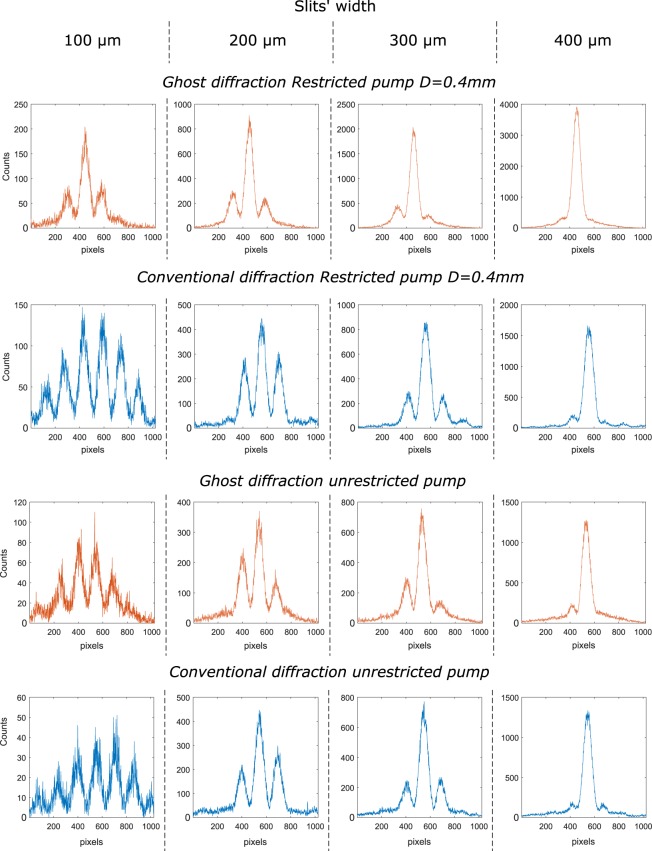
Ghost and conventional diffraction for different slit widths (respectively in orange and blue). The slit separation is in all cases 500 *μm* and the pump aperture is restricted to a diameter of D = 0.4 mm in the first two rows. The pump has a diameter of *D* = 2*w*_*p*_ = 0.9 *mm* in the two last rows, where *w*_*p*_ is the pump beam’s beam waist.

By using SLMs to display the slits, we can test a variety of different double-slit patterns. In order to test the limit of ghost diffraction and to compare it with conventional diffraction we have recorded the diffraction from the double-slits of various widths but with a constant separation of 500 *μm*. We have recorded the diffraction patterns for both ghost and conventional configurations. Figure 2 shows different interference patterns obtained through either ghost diffraction (orange curves) or conventional diffraction (blue curves) and when the aperture *A*_*p*_ of diameter D = 0.4 mm is optionally introduced in the pump to restrict its aperture.

We can see in Fig. 2 that both the ghost diffraction and the conventional diffraction exhibits an increased spread in the positions of the photons when the slits become smaller. The experimental observations decide therefore against Popper’s predictions^18^ and are in accordance with the Copenhagen interpretation. However, one can also observe in Fig. 2 that toward the small values of the slits width the extent of the ghost diffraction pattern is limited which is in contrast to the conventional diffraction that does not appear to be. This behaviour corresponds to the predictions of Collett and Loudon.

To assess more quantitatively this effect we have fitted the measured data with the expected function corresponding to the double slits diffraction pattern:

$$I={I}_{0}\cdot {{\rm{sinc}}}^{2}(\pi \cdot {w}_{a}\cdot x)\cdot {\cos }^{2}(\pi \cdot {w}_{b}\cdot x)$$

where x is the transverse position along the camera plane, *I*_0_ is a normalising intensity, *w*_*a*_ is the spatial frequency associated with the slits width a and *w*_*b*_ is the spatial frequency associated with the slit separation b. Our aim is to extract the values of *w*_*a*_ to characterise the diffraction associated with the slit width a. The theoretical prediction for the conventional diffraction of the slits is given by the following expression: ${w}_{a}=\frac{a}{{\rm{\lambda }}f}$ where *λ* = 710 *nm* is the wavelength of the diffracted light, *f* = 1333 *mm* is the effective focal length producing the Fourier transform from the SLMs planes to the camera plane and *a* is the width of the slit.

**Figure 3:**
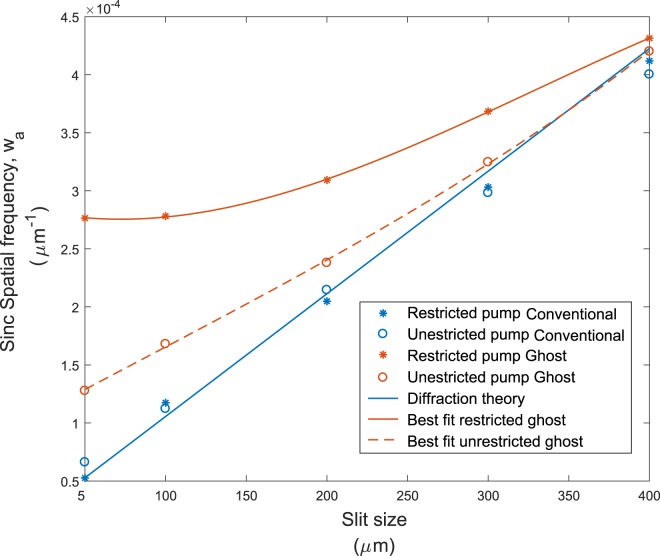
Spatial frequency of the sinc envelope of the diffraction patterns as function of the slits’ width. The orange asterisks correspond to the measured ghost diffraction using a pump beam with a restricted aperture. The orange circles correspond to measured ghost diffraction but with an unrestricted pump. The solid blue line corresponds to the theoretical prediction for an unrestricted diffraction. The blue asterisks and circles correspond to the measured conventional diffraction measurements, with both restricted and unrestricted pump. Error bars not shown here for clarity are estimated to be ±0.1 × 10^−4^ *μm*^−1^ along the y axis.

We show in Fig. 3 the values of the optimum fitting parameter *w*_*a*_ as a function of the slits width in various cases of restricted and unrestricted pump beam diameters. As expected we observe that, for both restricted and unrestricted pump beam, the form of the conventional diffraction follows the conventional theory (blue curve). Similarly, for an unrestricted pump beam we see that the ghost diffraction shows a similar result to that of the conventional diffraction. However, for a pump beam of reduced diameter, the extent of the diffraction is actually limited even when the slit width is made very small. As discussed above, we are able to show the two regimes for ghost diffraction. The first regime corresponds to the following observations: toward the large values for the slits’ width and with an unrestricted pump beam the ghost diffraction behaviour is similar to the conventional diffraction with perfect compatibility to the Copenhagen interpretation of quantum mechanics: when the ghost slit is made smaller we have more knowledge about the position of the twin photon and consequently its momentum spreads. However, the information about the ghost slit being opened or closed can only be accessed through harnessing the correlations and therefore the No-Communication theorem of quantum mechanics prevents superluminal communication to be established through this phenomenon. The second regime, toward the small slit width and a restricted pump beam, shows the ghost diffraction is not significant, in agreement with the theoretical description made by Collett and Loudon^12^. However, this observation still remains compatible with the Copenhagen interpretation as our ability to infer the momentum of one photon by detecting the other photon is limited by the transverse spatial extent of the correlations. In this case, when the slit is smaller than the correlation width, we do not increase our knowledge of the other photon position by decreasing the size of the slit and therefore as depicted in the Copenhagen interpretation there is no increase in the momentum spread of the photon in the other arm. Quantitative details are given in the supplementary material on this last matter.

## Discussion

The experimental observations reported in this work seem therefore to decide against Popper’s predictions and instead are fully in accordance with the Copenhagen interpretation. If an aperture is introduced on the path of one of the signal photons, then the subset of coincidently detected idlers photon in the second arm will reveal the diffraction pattern of the slit. However, it has also been observed that for slits smaller than the spatial extent of the correlations the ghost diffraction is limited which corresponds to Collett and Loudon predictions. Such a phenomenon is compatible with standard quantum mechanics predictions^12^, is also perfectly compatible with the Copenhagen interpretation of quantum mechanics.

An intuitive understanding of these observations can be drawn from a realisation that the observation of a ghost image or a ghost diffraction pattern is only apparent from a correlation between photon detections in the two beams, be they classical or quantum. Although the phenomenon presented in the present implementation uses an underlying spatial entanglement, it must be noted that no EPR or Bell behaviour can be deduced from the experiment suggested by Popper, as the photons are detected with a fixed measurement basis. Therefore, a local hidden variable theory would still predict the same observed results. In this sense the detection of an idler photon can be considered here as post-selecting a subset of signal photons and it is this subset that reveals the spatial form of the diffraction pattern. Given that the spatial pattern is formed from a subset of the signal photons, the spatial extent of the subset can never exceed the original distribution of photons. Effectively, the spatial extent of the recorded pattern can be no larger than the source (since the detector is in the image plane of the source) and the size of the source is itself limited by the size of the pump beam. Such a result, remains perfectly compatible with the Copenhagen interpretation of quantum mechanics.

To conclude, we have experimentally implemented Popper’s thought experiment by studying ghost diffraction and its limits through an self scaled experiment using double slit diffraction. We have explored here the full complexity of this experiment by distinguishing two regimes for wide and narrow slit sizes. This allowed us to discuss Popper’s experiment in its full complexity. Our results are perfectly compatible with the Copenhagen interpretation of quantum mechanics, but it should also be acknowledged that they shed no light on its validity compared to the many other interpretations.

## Electronic supplementary material

Supplementary material

## Data Availability

The datasets generated during and/or analysed during the current study are available in the University of Glasgow repository, 10.5525/gla.researchdata.659.
