# Supplementary material for: Experimental Limits of Ghost Diffraction: Popper’s Thought Experiment
Source: Sci Rep. 2018 Sep 4;8:13183. doi: 10.1038/s41598-018-31429-y (PMC6123420; doi:10.1038/s41598-018-31429-y)
Supplement: Supplementary file 1 — Supplementary material [file 41598_2018_31429_MOESM1_ESM.pdf]

Supplementary material, Experimental Limits of Ghost Diffraction : Popper's Thought Experiment

Paul-Antoine Moreau[1,*], Peter A. Morris[1], Ermes Toninelli[1], Thomas Gregory[1], Reuben S. Aspden[1], Gabriel Spalding[2], Robert W. Boyd[1,3,4], and Miles J. Padgett[1]

[1]School of Physics and Astronomy, University of Glasgow, G12 8QQ, UK
[2]Department of Physics, Illinois Wesleyan University, Bloomington, Illinois 61701, USA
[3]Department of Physics, University of Ottawa, Ottawa, Ontario, Canada
[4]The Institute of Optics and Department of Physics and Astronomy, University of Rochester, Rochester, NY 14627, USA
[*]Corresponding author: paul-antoine.moreau@glasgow.ac.uk

ABSTRACT

In this supplementary material we show quantitatively that the extent of the diffraction observed in the ghost diffraction patterns is limited by a size corresponding to the size of the spatial correlation in the diffracting object plane. Which is coherent with the observation made on Fig. 3 of the main article that in the case of the unrestricted pump (red circles) the ghost diffraction seems to follow more closely the conventional diffraction behavior, due to smaller correlations in the object plane. It is also makes the absence of diffraction in that regime compatible with the Copenhagen interpretation of quantum mechanics: When the size of the slits are smaller than the size of the correlation one cannot get anymore spatial information about the second photon localization in the SLM plane by narrowing even more the slits in the heralding photon path. One can indeed only infer the position of one of the photons with the information on the other photon position with an accuracy that is limited by the size of the correlations.

We first calculate the size of the correlations obtained in the SLMs planes in the case of the pump restricted by the aperture A_p,with the aim to compare this value to the limit diffraction limit for ghost diffraction obtained in the same conditions. The limiting factor for the size of the correlations in the far field of the crystal is the diameter of the pump that is set experimentally by the size of the aperture A_p that has a diameter D=0.4mm. We can use a Gaussian approximation to evaluate the spatial strength of the correlations in the Far-field i.e. in the planes of the SLMs:

$$w_c^{th} = f\frac{4\lambda_p}{\pi D} \tag{1}$$

Where f=100mm is the effective focal of the imaging system producing the Fourier transform and $\lambda_p = 355nm$ is the pump wavelength. We can therefore expect spatial correlations with a radius of $w_c^{th} = 113\mu$m in the plane of the camera SLM. This value has been confirmed experimentally by imaging the SLM plane with the camera using the narrowest accessible slit size for photon 1 and acquiring the ghost image that then corresponds to the correlations. We have obtained experimentally a correlation peak of size $w_c^{exp} = 106 \pm 10\mu m$ in good agreement with the predicted value.
One can now compute the size of the diffraction pattern obtained in the camera plane due to the diffraction of the correlations feature sizes $w_c^{th} = 113\mu$m. Again, using a Gaussian approximation we obtain a gaussian envelope diffraction in the camera plane of size:

$$w_g^{th} = f\frac{2\lambda_p}{\pi w_c^{th}}, \tag{2}$$

With $f = 1333.3mm$. This gives a value of $w_g^{th} = 2.67mm$.
Now, our aim is to show that this size is what limits of the ghost diffraction spread toward the small slit width (observed on the red point of Figure 3). We have fitted the ghost interference pattern obtained with a slit width of 100 microns with a Gaussian instead of a cardinal sine. A gaussian profile of type $\exp(-2x^2/\,{w_g}^2)$ is closer to the correlation shape than a sinc. We find $w_g = 2596 \pm 100\mu m$ This Gaussian can be propagated to the SLM far field plane:

$$w_{g(slm)} = f\frac{2\lambda_p}{\pi w_g}, \tag{3}$$

Where $f = 1333.3mm$ is the effective focal of the imaging system producing the Fourier transform.

We find $w_{g(slm)} = 116 \pm 5\mu m$ which is in good agreement with both the theoretical ($w_c^{th} = 113\mu m$) and experimental ($w_c^{exp} = 106 \pm 10\mu m$) values obtained above for the size of the correlations. The size of the correlations in the diffractive object plane is therefore the characteristic size that limits the observed ghost diffraction phenomenon.
